# Plant homeodomain proteins provide a mechanism for how leaves grow wide

**DOI:** 10.1242/dev.193623

**Published:** 2020-10-21

**Authors:** Phillip A. Conklin, Robyn Johnston, Brianne R. Conlon, Rena Shimizu, Michael J. Scanlon

**Affiliations:** 1 Plant Biology Section, School of Integrative Plant Science, Cornell University, Ithaca, NY 14853, USA; 2 The Elshire Group Limited, Palmerston North 4472, New Zealand

**Keywords:** Maize, Leaf, Narrow sheath, Homeobox, Margin

## Abstract

The mechanisms whereby leaf anlagen undergo proliferative growth and expansion to form wide, flat leaves are unclear. The maize gene *NARROWSHEATH1* (*NS1*) is a *WUSCHEL-related homeobox3* (*WOX3*) homolog expressed at the margins of leaf primordia, and is required for mediolateral outgrowth. To investigate the mechanisms of NS1 function, we used chromatin immunoprecipitation and laser-microdissection RNA-seq of leaf primordial margins to identify gene targets bound and modulated by NS1. Microscopic analyses of cell division and gene expression in expanding leaves, and reverse genetic analyses of homologous NS1 target genes in *Arabidopsis*, reveal that NS1 controls mediolateral outgrowth by repression of a growth inhibitor and promotion of cell division at primordial leaf margins. Intriguingly, homologous WOX gene products are expressed in stem cell-organizing centers and traffic to adjoining cells to activate stem-cell identity non-autonomously. In contrast, WOX3/NS1 does not traffic, and stimulates cell divisions in the same cells in which it is transcribed.

## Abstract (precis)

**Highlighted Article:** The NS1 homeodomain transcription factor regulates lateral organ outgrowth from shoot meristems and leaf primordial margins by repressing the expression of negative growth regulators.

## INTRODUCTION

Plant leaves are typically dorsiventrally flattened and broad, to maximize light capture, gas exchange and photosynthetic efficiency. Leaves develop from the periphery of shoot apical meristems (SAMs), which comprise pools of pluripotent stem cells that give rise to all the above-ground organs of the plant. Leaf primordia are dorsiventrally asymmetrical from their inception (Kaplan, 2001; Caggiano et al., 2017); the dorsal side of the primordium develops adjacent to the SAM, and receives molecular signals that are distinct to those of the ventral side of the newly-emerged leaf. A mechanistic model for leaf outgrowth and flattening, inspired by molecular-genetic analyses of organ development in animals, proposed that the juxtaposition of dorsal and ventral leaf domains at the pre-primordial leaf margin organizes outgrowth along the mediolateral and proximodistal axes to generate wide leaves that project out from the stem (Waites and Hudson, 1995; Diaz-Benjumea and Cohen, 1993; Williams et al., 1994). Although several decades of molecular genetic analyses provide widespread support for this model, the detailed mechanisms whereby plant leaves grow wide remain unclear.

**Fig. 1.**
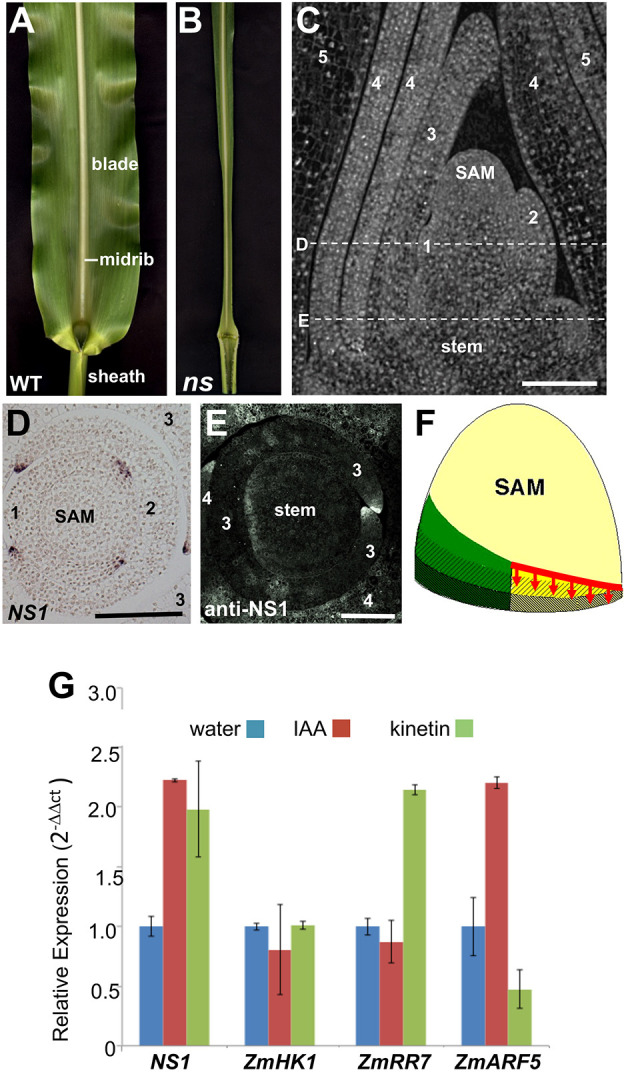
**The maize duplicate factor homeobox genes *NS1* and *NS2* are required for mediolateral outgrowth of leaf primordia.** (A,B) Wild-type sibling (*NS1/ns1-R; ns2-R/ns2-R*) (A) and *ns* (*ns1-R/ns1-R; ns2-R/ns2-*R) adult (B) leaves; the *ns* double mutant has narrow leaves that fail to expand mediolaterally. (C) Computed tomographic (CT) image of a wild-type 14 day seedling shoot apex from the maize inbred B73 (*NS1;NS2*) showing the shoot apical meristem (SAM) surrounded by five successive leaf primordia numbered according to plastochron #1-5, wherein plastochron 1 is the newest leaf to arise from the SAM. The dashed lines show the approximate locations of transverse sections depicted in D and E. (D,E) *NS1 in situ* hybridization (D) and immunohistochemistry using an anti-NS1 antibody (E) shows that *NS1* gene products accumulate in the margins of leaf primordia and in the pre-primordial margins of the plastochron 1 leaf in D, before the margins emerge from the SAM. (F) A model wherein the maize phytomer (leaf and stem) is formed from the SAM (tan color) and comprise at least two distinct developmental compartments that extend along three proximodistal domains corresponding to the distal leaf blade (unhatched), the proximal leaf sheath (hatched right to left) and the stem (hatched left to right). The green regions depict the central compartment adjacent to the midrib, which contains phytomer domains that are present in both wild-type and *ns* mutant leaves. The yellow region shows the lateral domains, which requires NS function at the pre-primordial and primordial leaf margin (red line) to grow out from the SAM and expand the leaf primordium mediolaterally. (G) *NS1* transcript accumulation is induced in maize seedlings following application of the phytohormones auxin and cytokinin. Controls include maize homologs of *HK1* (not transcriptionally-induced by cytokinin), *ARR7* (induced by cytokinin) and *ARF5* (induced by auxin). CT image in C provided by S. Leiboff (Cornell University, USA). *In situ* hybridization in D provided by D. Henderson (University of Georgia, USA). Data are mean±s.e.m. Scale bars: 50 µm.

Duplicate mutations in the maize *WUSCHEL-related homeobox3* (*WOX3*) genes *NARROWSHEATH1* (*NS1*) and *NARROWSHEATH2* (*NS2*) cause narrow leaves that fail to expand mediolaterally (Fig. 1A,B; Scanlon et al., 1996). Although the distal-most blade and leaf domains adjacent to the midrib are both intact in mature *ns* mutant (*ns1-R ns2-R*) leaves, lateral leaf domains are absent from the proximal blade and the entire length of the sheath. Predicted to encode transcription factors, *NS1* and *NS2* transcripts and protein accumulate in the margins of leaf primordia, and in the pre-primordial margins of leaf founder cells before they grow out from the SAM (Fig. 1C-F; Fig. S1; Nardmann et al., 2004; Shimizu et al., 2009). These phenotypes suggested a model wherein maize leaves comprise at least two distinct developmental compartments; the central compartment adjacent to the midrib contains blade and sheath domains that are present in both wild-type and *ns* mutant leaves (green regions in Fig. 1F), whereas the lateral domain requires NS function to grow out from the SAM and expand the leaf mediolaterally (yellow regions in Fig. 1F; Scanlon et al., 1996; Scanlon, 2000). Likewise, mutations in homologous WOX genes in *Arabidopsis*, *Nicotiana*, *Medicago*, *Petunia* and rice condition similar narrow leaf and lateral organ phenotypes, and their wild-type expression patterns overlap in the margins of incipient and emerged leaf primordia (Fig. S1; Matsumoto and Okada, 2001; Vandenbussche et al., 2009; Tadege et al., 2011; Nakata et al., 2012; Cho et al., 2013). These phenotypic and expression data suggest that specific plant homeobox genes are required for mediolateral and proximodistal outgrowth from the juxtaposed, dorsal and ventral domains at leaf primordial margins (Fig. 1F), as predicted by the Waites and Hudson (1995) model. However, the mechanisms whereby these leaf-specific WOX genes function during leaf initiation and expansion are unknown.

Here, we report the use of chromatin immunoprecipitation sequencing (ChIP-seq) and laser microdissection RNA sequencing (LM-RNA-seq) to identify genes bound and modulated by the maize homeodomain protein NS1/WOX3. Comparative reverse genetic analyses of homologous gene targets, combined with molecular genetic and microscopic examinations of leaf margin development suggest a model whereby plant homeobox genes make leaves grow wide.

## RESULTS

### NS1/ functions downstream of auxin

The phytohormone auxin is a conserved regulator of leaf initiation; transport-induced auxin maxima in the SAM epidermis correlate with the sites of new primordial outgrowth in diverse plant species (Reinhardt et al., 2000; O'Connor et al., 2014). Accumulation of *NS1* transcripts is upregulated more than twofold after application of 0.1 µM auxin (indole acetic acid) to maize seedlings (Fig. 1G); equivalent upregulation is observed following treatment with 0.1 µM cytokinin (kinetin). The *Arabidopsis WOX3* ortholog *PRS1* is also upregulated ∼twofold by auxin treatment (Caggiano et al., 2017), suggesting that both of these orthologous leaf homeobox genes act downstream of auxin. Moreover, comparisons of wild-type and *ns* mutant seedlings revealed no changes in transcript accumulation for *SPARSE INFLORESCENCE1* (*SPI1*), the maize homolog of the *Arabidopsis* auxin biosynthetic gene *YUCCA 1* (Fig. S2A-D; Gallavotti et al., 2008; Zhao et al., 2001). In addition, localization of the DR5∼RFP auxin-response reporter and accumulation of PIN1-like auxin transport proteins are equivalent in the margins of *ns* mutant and wild-type sibling leaf primordia (Fig. S2E-J). Taken together, these data suggest that NS1 functions downstream of auxin biosynthesis, transport, and response.

### Identification of gene targets bound and modulated by NS1

**Fig. 2.**
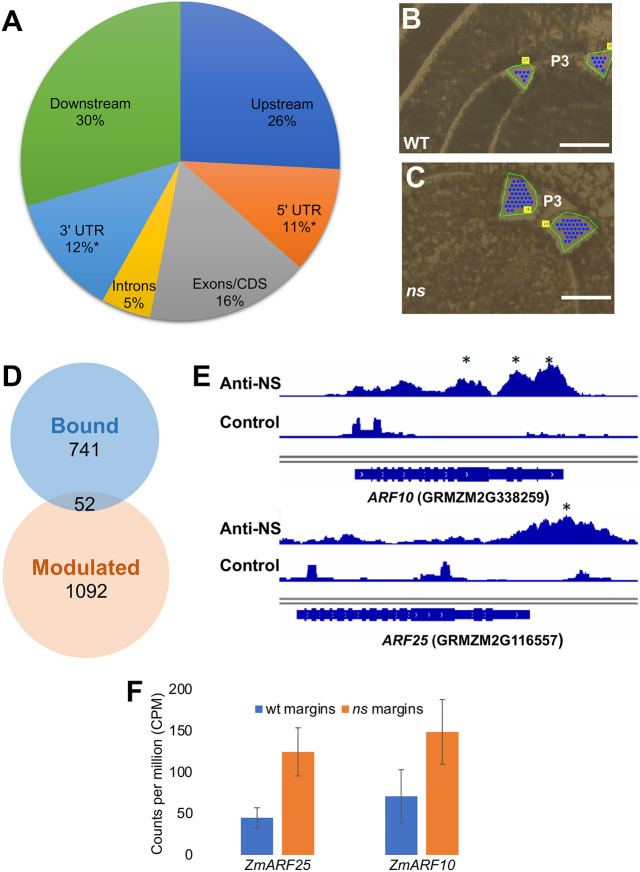
**Gene targets bound and modulated by NS1.** (A) Distribution of significant genomic DNA targets bound by NS1 protein in ChIP-seq analyses; asterisk represents peak enrichment in genomic regions, *P*-value<0.05 (binomial test of peak enrichment). Upstream and Downstream refer to the position of NS1-bound genomic sequences relative to the nearest predicted gene model. (B,C) Representative selected tissue for margins microdissected for RNA-seq analyses from wild-type (WT) sibling (B) and *ns* mutant (C) leaf primordia. Green line indicates outline of leaf primordium before microdissection; blue dots indicate areas of laser pulses, focused underneath the leaf tissue, used to catapult the targeted tissue off of the slide; numbers in yellow boxes are the sample numbers generated by the PALM microlaser system, as described in Scanlon et al. (2009). (D) Venn diagram showing bound and modulated gene targets of NS1. (E) Integrative Genomics Viewer (IGV) tracks showing peaks for NS1 binding to genomic DNA near the 3′ regions of *ARF10* and *ARF25* gene targets; asterisk represents peaks with a *q*-value<0.05 (binomial test of peak enrichment). (F) LM-RNA-seq data showing the accumulation of *ARF25* and *ARF10* transcripts in wild-type sibling and *ns* mutant leaf margins. Data are mean±s.e.m. Scale bars: 50 µm.

An NS1 polyclonal antibody described in Shimizu et al. (2009) identifies leaf homeodomain protein accumulation in approximately three cells at the margins of leaf primordia, and in pre-primordial margins of the incipient leaf primordium before it emerges from the SAM (Fig. 1E; Fig. S1A-F). Chromatin from two-week-old B73 seedlings dissected to contain meristematic and young leaf tissue were used in a ChIP-seq experiment. Comparisons of NS1-targeted genomic sequences versus those bound by the non-specific control antibody found that NS1 bound to a total of 2518 loci found with a *q*-value cut-off of 0.5, which corresponds to 793 nearest genes. We found that 80.4% of the peaks were within 10 kb of genes, with the furthest being 386,940 bps away (Table S1). Bound sequences were significantly enriched in 5′ untranslated regions (UTRs) (271 peaks or 10.8% FDR 0.0046) and 3′UTRs (311 peaks or 12.4% FDR 0.00017) (Fig. 2A).

To identify genes that are both bound and transcriptionally modulated by NS1, LM-RNA-seq was used to harvest tissue and extract RNA from the marginal tips of the second and third leaf primordia closest to the SAM (i.e. P2 and P3 staged leaves), containing the cells where *NS1* transcripts accumulate (Fig. 2B,C). RNA-seq of these microdissected margin cells identified 1144 genes that are differentially expressed (DE) in *ns* mutant primordial margins (Fig. 2D; Table S2). Union of the 793 genes bound by NS1 and the 1144 transcripts DE in ns margins identified a total of 52 genes that are bound and modulated by the NS1 homeodomain transcription factor. The majority of NS1 bound-and-modulated genes (36/52) were transcriptionally repressed (Table S3); these data are consistent with previous reports that the WUSCHEL-class of WOX transcription factors function predominantly as transcriptional repressors (Lin et al., 2013), although some NS1 transcriptional target genes are indeed activated (Leibfried et al., 2005; Busch et al., 2010; Yadav et al., 2013; Pi et al., 2015). The top 11 genes with the most-enriched ChIP-seq peaks of transcriptionally-repressed NS1 target genes all have peaks within the transcriptional termination site or the last exon (Table S4), and include the predicted transcription factors *ETHYLENE RESPONSE FACTOR 7* and *ERF DOMAIN PROTEIN 9*, *HAIRY MERISTEM 1*, *JASMONATE-ZIM-DOMAIN PROTEIN 1*, and two maize paralogs of the *Arabidopsis AUXIN RESPONSE FACTOR 2* (*ARF2*) gene (*ARF10* and *ARF25*; Galli et al., 2018). Intriguingly, *ARF2* is previously described as a repressor of lateral organ growth in *Arabidopsis*; *arf2* mutations condition enlarged leaf lamina (Okushima et al., 2005; Schruff et al., 2006).

### Analyses of *ARF2* homologs in maize and *Arabidopsis*

**Fig. 3.**
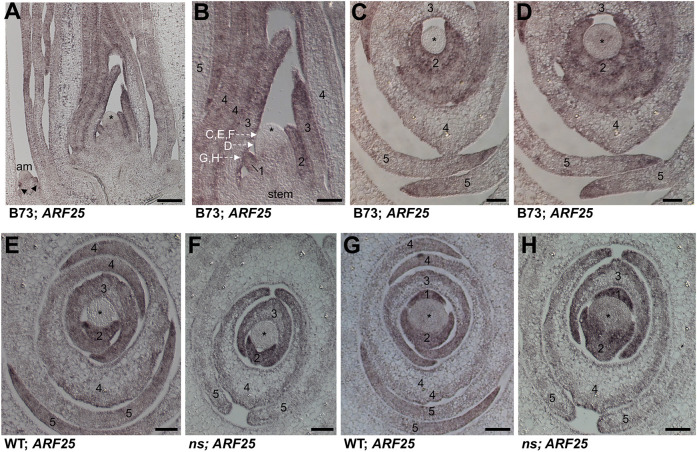
***In situ* hybridization of maize seedlings using an *ARF25* probe.** (A,B) Longitudinal section through shoot apex and axillary meristem (am) of a wild-type B73 seedling. B shows enlargement of A. Dashed arrows designate the approximate proximodistal locations of transverse sections through the shoot apex shown in panels C-H. Black arrowheads indicate husk leaf primordia produced by the axillary meristem. (C,D) Transverse section through the shoot apex of a wild-type B73 seedling. (E-H) Transverse sections through the shoot apices of wild-type sibling (E,G) and *ns* mutant (F,H) seedlings; genotypes are as described in Fig. 1*.* Asterisks indicate SAM; numbers indicate plastochron numbers of leaf primordia as in Fig. 1. Blue-brown signal indicates transcript accumulation of *ARF25* and its paralog *ARF10*. Scale bars: 100 µm (A,B); 50 µm (C-H).

*ARF10* and *ARF25* comprise duplicated maize orthologs of *Arabidopsis* *ARF2* that are significantly bound and modulated by NS1 (Fig. 2E). *ARF10* and *ARF25* have a peak fold enrichment of 7.77 (9.69E-11) and 8.32 (*q*-value 4.36E-10), respectively. LM-RNA-seq analyses of the marginal tips of P2-P3 leaf primordia reveal that *ARF10* and *ARF25* are 2.1- and 2.7-fold higher (adjusted *P*-value of 2.37E-2 and 1.43E-4, respectively) in *ns* mutant leaf margins when compared with wild-type siblings (Fig. 2F; Table S2). Owing to 90.7% nucleotide identity among their coding sequences, paralog-specific nucleic acid hybridization probes cannot be constructed. In agreement with previous transcriptomic analyses showing widespread expression of *ARF10* and *ARF25* during maize ontogeny (Harper et al., 2011), *in situ* hybridization analyses reveal that *ARF10/ARF25* transcripts accumulate throughout the maize seedling shoot (Fig. 3; Knauer et al., 2019). *ARF10/ARF25* transcripts are found throughout young leaf primordia, including the husk leaves of axillary meristems, and are enriched in the epidermis and margins of later primordia (Fig. 3A). We note that *in situ* hybridizations are not inherently quantitative assays; no differences in *ARF10/ARF25* hybridization intensity are obvious in *ns* mutant and wild-type leaf primordia.

**Fig. 4.**
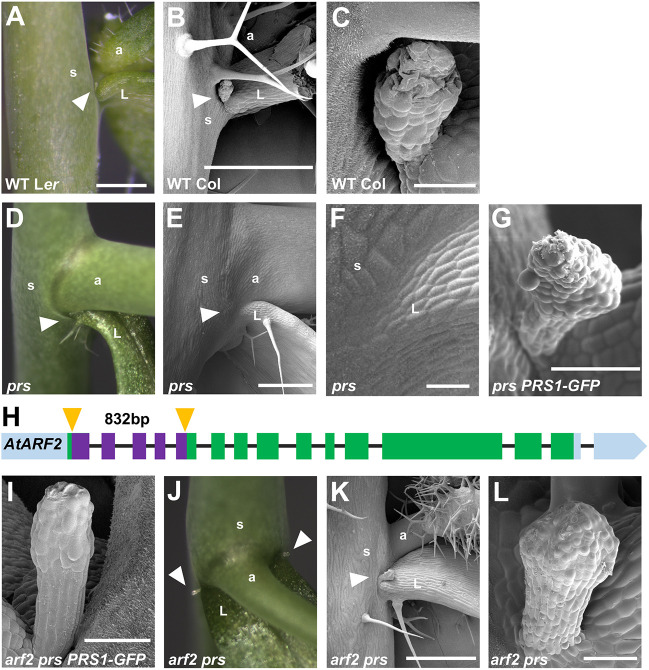
**The *Arabidopsis* arf2 mutation suppresses the lateral stipule deletion phenotype in *prs1* mutant leaves.** (A) Wild type (WT) stipule at the base of the cauline leaf in the Landsberg *erecta* (L*er*) background. (B,C) Scanning electron cryomicrograph (CryoSEM) of a WT stipule in the Columbia (Col) ecotype. (D) *prs* mutant lacks stipules at the base of the cauline leaf. (E,F) CryoSEM of *prs* mutant leaf attachment point. (G) CryoSEM of restored stipule in *prs PRS1-GFP* complemented plants. (H) *Arabidopsis* *ARF2* gene model. The *arf2-12* mutant allele contains an 832 bp deletion, depicted by purple boxes, with the CRISPR/Cas9 cut sites highlighted by yellow arrowheads. Green boxes represent exons; black line represent introns; blue boxes represent the 5′ and 3′ UTRs. (I) CryoSEM of *arf2* single mutant in the *prs PRS1-GFP* complementation background. (J) *arf2 prs1* double mutant stipules. (K,L) CryoSEM of restored stipules in *arf2 prs1* double mutants. White arrowheads indicate stipules. a, axillary branch; L, leaf; s, stem. Scale bars: 500 µm (A,B,E,K); 50 µm (C,F,G,I,L).

Two previous studies reported that *arf2* null mutations in *Arabidopsis* condition enlarged seeds and lateral organs, including leaves, stems and carpels, which is attributed to increased cell division and expansion. These data suggested that ARF2 functions as a pleotropic inhibitor of lateral organ growth in *Arabidopsis* (Okushima et al., 2005; Schruff et al., 2006). We next exploited this plant model system to determine whether *WOX3* and *ARF2* interact genetically in *Arabidopsis*, as predicted by our ChIP-seq analyses of NS1 function in maize. Single mutations in *PRS1/WOX3* cause either the complete deletion of the lateral sepals or extreme reductions in lateral sepal width (Fig. S3; Matsumoto and Okada, 2001). No defects in mediolateral development of the leaf lamina or petiole are described in *prs1/wox3* mutants, although the lateral stipules are completely deleted from the very base of *prs1/wox3* mutant leaves (Fig. 4A-G; Nardmann et al., 2004; Shimizu et al., 2009; Matsumoto and Okada, 2001). All floral and vegetative wild-type phenotypes are restored in *prs1/wox3* mutant plants via the introduction of the *PRS1-GFP* reporter allele driven by the native *PRS1* promoter (Fig. 4G; Fig. S3F; Shimizu et al., 2009). A novel *arf2* null allele (*arf2-12*) was generated by CRISPR/Cas9 mutagenesis, which contained an 832 bp deletion at the beginning of the coding sequence (Fig. 4H). In the complemented *prs1/wox3*; *pPRS1-PRS1*∼GFP background, *arf2-12* mutants show stereotypical *arf2* mutant phenotypes including elongated carpels and overgrowth of leaf laminar tissues; lateral stipules are intact at the leaf base (Fig. 4I; Fig. S3G). Moreover, the lateral stipules are restored in *arf2-12 prs1/wox3* double mutants (Fig. 4J-L), thereby suppressing the *prs/wox3* mutant leaf phenotype. In contrast, no rescue of the *pr1s/wox3* lateral sepal development phenotype is observed in *arf2-12 prs1/wox3* double mutant flowers (Fig. S3C,H).

### NS1 function activates cell division and growth from leaf margins

**Fig. 5.**
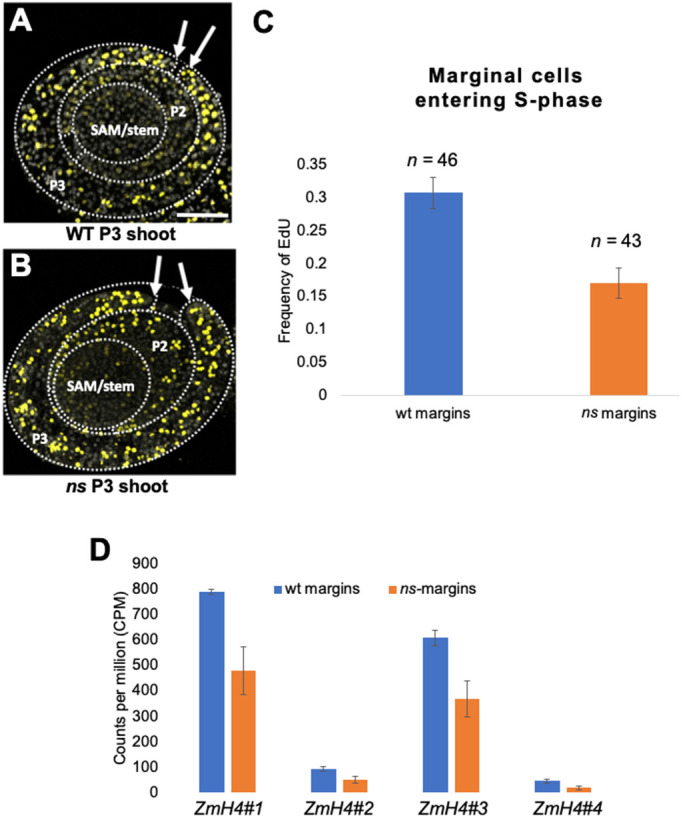
**NS1 promotes entry into S-phase in the margins of young leaf primordia.** (A,B) EdU-stained transverse sections were imaged for wild-type sibling (A) and *ns* mutant (B) P3 shoots containing the SAM and three leaf primordia (genotypes as described in Fig. 1). Arrows indicate the edges of P3 leaf margins. Dotted lines indicate the outlines of P3 and P2 leaf primordia, and SAM as labeled. (C) Quantification of the three most-marginal leaf primordial cells of the plastochron 3 leaf primordium entering S-phase within 3 h. (D) Counts per million of *ZmH4* transcripts significantly differentially expressed in wild-type sibling (blue) and *ns* mutant (orange) margins. *ZmH4* paralogs #1-#4 are GRMZM2G073275, GRMZM2G 084195, GRMZM2G421279, and GRMZM2G072855, respectively*.* Data are mean±s.e.m. Scale bar: 50 µm.

Genetic and molecular evidence suggests that NS1/WOX3 functions to promote cell proliferation at developing leaf margins (Scanlon et al., 1996; Scanlon, 2000; Nardmann et al., 2004). In a test of this model, incorporation of the thymidine analog 5-ethynyl-2′-deoxyuridine (EdU) was quantified in marginal cells of P3 wild-type and *ns* mutant leaf primordia, as a molecular marker for entry into the DNA synthesis phase (S-phase) of the cell cycle (Kaiser et al., 2009). As shown in Fig. 5A-C, the three cells at the tips of wild-type P3 leaf margins, where *NS1* transcripts and protein accumulate (Fig. 1D,E), enter the cell cycle approximately twice as frequently as cells in *ns* mutant P3 margins [counting wild-type margin pairs (*n*=46) and ns margin pairs (*n*=43) resulted in 1.8-fold higher frequency with a *P*-value of 0.0001]. Likewise, *HISTONE H4* (*H4*) expression comprises an additional marker for entry into S-phase in plant cells (Bilgin et al., 1999). Of the four *ZmH4* paralogs that are differentially expressed in *ns* mutant margins, our RNA-seq data reveal that they are all significantly downregulated in *ns* mutant P2/P3 leaf margins (i.e. GRMZM2G073275 1.75-fold, *P*-value=0.024; GRMZM2G084195 2.02-fold, *P*-value=0.033; GRMZM2G421279 1.76-fold, *P*-value=0.022; GRMZM2G072855 2.46-fold, *P*-value=0.028; Fig. 5D; Table S2). These data support the hypothesis that NS1 function promotes entry into S-phase in the marginal tip cells of maize leaf primordia.

**Fig. 6.**
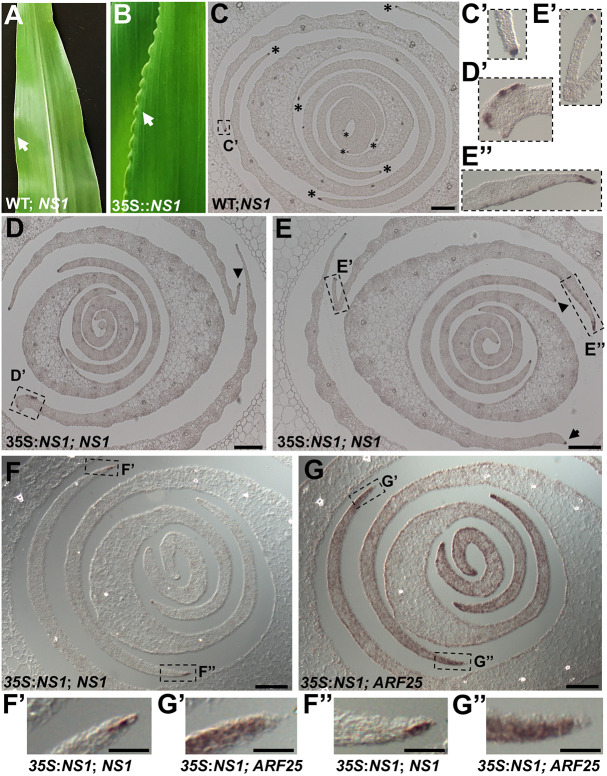
**NS1 overexpression promotes abnormal growth in leaf margins.** (A) Wild-type sibling (i.e. no 35S:NS1 construct) maize leaf. (B) Overgrowth at leaf margins in 35S*:NS1* maize leaf. White arrows indicate leaf margins. (C) *In situ* hybridization using *NS1* probe of a transverse section above the shoot apex of a wild-type sibling seedling. Asterisks indicate accumulation of *NS1* transcript at leaf margins. (C′) 4× magnification of boxed inset shown in C. (D,E) *In situ* hybridization using *NS1* probe of two different transverse sections above the shoot apex of a *35S::NS1* seedling. Arrow indicates normal, wild-type pattern of *NS1* transcript accumulation. Arrowheads and boxed insets indicate abnormal patterns of ectopic *NS1* transcript accumulation. (D′,E′,E″) 2.5× magnifications of boxed insets shown in D and E. (F,G) *In situ* hybridizations of adjacent, successive sections of a non-phenotypic 35S:*NS1* seedling hybridized to *NS1* (F) and *ARF25* probes (G). (F′,F″,G′,G″) 2.5× magnifications of boxed insets shown in F and G. Scale bars: 100 µm (C-G); 50 µm (F′,F″,G′,G″).

Moreover, transgenic maize plants overexpressing *NS1* from the constitutive 35S cauliflower mosaic virus (35S CaMV) promoter can exhibit abnormal proliferative outgrowths at leaf blade margins (Fig. 6A,B). Notably, these ruffled margin phenotypes are not found on all leaves of 35S:*NS1* transgenic plants and, when present, form only in the distal domains of the leaf blade that are unaffected by mutations in *NS1* and *NS2* (Scanlon et al., 1996). Surprisingly, *in situ* hybridizations of 35S:*NS1* transgenic leaves and wild-type siblings did not reveal constitutive accumulation of *NS1* transcripts throughout the leaf primordia of *NS1* overexpressing plants. In contrast, small patches of ectopic *NS1* transcript accumulation are observed in the distal regions of some transgenic leaf primordia; in all observed cases, these ectopic patches of maize *NS1*/*WOX3* homeobox gene expression correlated with abnormal thickening growth and/or elaborative outgrowth (i.e. ruffling) at or near the leaf margins (Fig. 6C-E′).

Lastly, *in situ* hybridizations were performed using *NS1* or *ARF25* probes on adjacent 10 µm histological sections of non-phenotypic plants harboring the 35S:*NS1* overexpression construct (Fig. 6F,G″). Thus, these samples had wild type-like leaf margin phenotypes and showed no evidence of ectopic expression of *NS1*, in spite of the fact that they contained the 35S:*NS* transgene. Comparisons of adjacent sections predominately showed that the extreme marginal tip cells of these leaf primordia exhibit complementary expression of *NS1* and *ARF10/ARF25*. That is, the leaf marginal tips accumulated *NS1* transcripts (Fig. 6F′,F″) but were typically free of detectable *ARF10/ARF25* mRNA (Fig. 6G′,G″), although *ARF10/ARF25* expression is detected in the submarginal regions of these same leaves (Fig. 6G-G″).

## DISCUSSION

An abundance of evidence supports the hypothesis that NS1/WOX3 promotes mediolateral outgrowth of leaves by the activation of cell proliferation at developing leaf margins. This evidence includes: the *ns1* mutant phenotype (Fig. 1A,B; Scanlon et al., 1996); accumulation of NS1 at leaf primordial margins (Fig. 1D,E; Nardmann et al., 2004); NS1 repression of the putative growth-repressing *Arabidopsis* *ARF2* homologs *ARF10* and *ARF25* (Fig. 2G); suppression of the *Arabidopsis prs1/wox3* lateral stipule deletion phenotype by the *arf2-12* mutation (Fig. 4J-L); elevated entry into S-phase by *NS1*-expressing leaf marginal cells (Fig. 5); and proliferative outgrowth of maize leaf margins overexpressing *NS1* (Fig. 6C-E). Our ChIP-seq analysis suggests that NS1 activates leaf outgrowth indirectly, at least in part via the direct transcriptional repression of maize orthologs of the *Arabidopsis* growth repressor *ARF2*. Although activation via ‘repression of a repressor function’ may seem like a non-intuitive mechanistic strategy, this phenomenon is replete with examples from animal and plant development including previously described WOX gene functions (Leibfried et al., 2005; Boyer et al., 2005; Loh et al., 2006; Yadav et al., 2013; Pi et al., 2015). For example, the *Arabidopsis* homeodomain proteins WOX5 and WUS1 maintain stem cell identity in shoot and root meristems, respectively, by actively repressing transcription of target genes that promote differentiation programs. Our data reveal that, like WUS1 (Busch et al., 2010; Yadav et al., 2013), NS1/WOX3 functions as both a repressor and activator of target gene expression, in contrast with previous reports that WUS-class WOX genes function solely as transcriptional repressors (Lin et al., 2013). The rescue of the *prs1/wox3* lateral stipule deletion phenotype in *arf2 prs1* double mutants suggests that a genetic interaction between WOX3 and ARF2 identified herein is conserved in *Arabidopsis* and maize, although we currently have no evidence that the PRS1/WOX3 transcription factor directly represses *ARF2* transcription. However, the *prs1/wox3* lateral sepal deletion phenotype is not suppressed by the *arf2* mutation (Fig. S3), suggesting that WOX3 function in *Arabidopsis* involves direct regulation of some additional gene target(s) aside from *Arabidopsis* *ARF2.* Reverse genetic analyses, in both maize and *Arabidopsis*, of additional NS1/WOX3 targets identified in this study (Table S3) will enable in-depth analyses of conserved and non-conserved WOX3 function in these model angiosperms.

Previous work in *Arabidopsis* reported that *PRS1/WOX3* (as well as the leaf homeobox gene *WOX1*, which has no ortholog in maize) is transcriptionally activated by the adaxially-localized protein ARF5, and is repressed by the abaxial transcription factors ARF3, ARF4 and ARF2 (Guan et al., 2017). In this way, PRS1/WOX3 and WOX1 accumulation is localized to the leaf primordial margin, at the juxtaposition of adaxial and abaxial leaf domains. Although it is not known whether PRS1/WOX3 repression of *ARF2* expression is conserved in *Arabidopsis* as well as in maize, *arf2* mutations suppress the *prs1* mutant leaf phenotype (Fig. 4J-L), revealing a genetic interaction. Future ChIP-seq analyses of PRS1/WOX3 will determine whether ARF2 and PRS1 are indeed mutually-repressive.

*NS1* is transcriptionally induced by both indole acetic acid (IAA) and kinetin (Fig. 1G), suggesting that NS1 function is downstream of auxin and cytokinin. We propose that NS1 is involved in lateral organ outgrowth, which is likewise associated with auxin (Reinhardt et al., 2000), and that this leaf outgrowth is mediated by cytokinin-activated cell divisions of organ initial cells at primordial leaf margins (Fig. 5). Furthermore, auxin accumulation during leaf initiation causes downregulation of *KNOTTED1*-like HOMEOBOX (KNOX) genes in leaf founder cells (Scanlon, 2003; Hay et al., 2006), although the detailed mechanism is unknown. Intriguingly, KNOX gene downregulation is incomplete in *ns* mutant SAMs, which correlates with the failure to elaborate lateral leaf domains (Scanlon et al., 1996). Although KNOX downregulation is disrupted in *ns* mutants, our data reveal that transcript accumulation of the maize auxin biosynthetic gene *SPI1*, accumulation of PIN1c auxin transport protein and localization of the DR5 auxin response reporter are not disrupted in *ns* mutant shoot apices (Fig. S2). These data implicate loss of NS1 function, and not defects in auxin biology per se, as responsible for the altered KNOX downregulation in *ns* mutant SAMs. Moreover, transcription of *NS1* is activated by auxin (Fig. 1G), although our ChIP-seq data suggest that KNOX genes are not targeted by the NS1 transcription factor (Table S1). Taken together, these data suggest that NS1 acts downstream of auxin in a network to downregulate KNOX accumulation in maize founder cells, although the role of NS1 during KNOX downregulation is indirect.

We note that the restriction of *NS1* ectopic overexpression to relatively infrequent small patches of transcript accumulation when driven by the constitutive 35S CMV promoter (Fig. 6D,E) reveals that maize has evolved an extraordinarily robust mechanism to confine *NS1* gene expression to the marginal tips of leaf primordia. AUXIN RESPONSE FACTORS and *WOX3* homologs in maize and *Arabidopsis* are upregulated by auxin (Fig. 1G; Caggiano et al., 2017; Galli et al., 2018; Ori, 2019). However, whereas *ARF10* and *ARF25* transcripts accumulate broadly in maize primordia (Fig. 3) but are reduced within the edges of leaf margins (Fig. 6G), *NS1* expression is limited to a few cells at the marginal leaf tips (Fig. 1D; Fig. 6C,F; Nardmann et al., 2004). These data suggest a model wherein auxin induces expression of *ARF10*, *ARF25* and *NS1* in maize leaf primordia. Thereafter, NS1 accumulation is restricted to the marginal tip cells by some unknown factor(s), and represses the expression of growth-inhibitory *ARF2* homologs in these same *NS1-*expressing cells at the leaf tip. In this way, auxin-induced NS1/WOX3 function promotes mediolateral expansion from the leaf margin. The well-studied WOX homeodomain proteins WUS and WOX5 traffic from the stem-cell organizing centers that express their corresponding mRNAs, to specify stem-cell identity in neighboring cells of the shoot and root meristem, respectively (Pi et al., 2015; Yadav et al., 2011). In contrast, our previous studies showed that PRS1/WOX3 does not traffic (Shimizu et al., 2009); this current study suggests that the leaf homeobox gene *NS1/WOX3* has evolved to activate cell division and proliferative growth in the same leaf margin cells in which it is expressed.

## MATERIALS AND METHODS

### Genetic stocks and plant growth

Maize stocks segregating for the narrow sheath mutant phenotype were obtained from the ns 1:1 line as previously described (Scanlon et al., 1996); phenotypically wild-type plants from this line are heterozygous for the *ns1* mutation and homozygous for the *ns2* mutation (genotype *NS1/ns1-R ns2-R/ns2-R*), whereas *ns* mutant plants are homozygous for both *ns1-R* and *ns2-R*. To generate the 35S:NS1 overexpression lines, the *NS1* (GRMZM2G069028) coding sequence was cloned into the entry vector pENTR/D (Thermo Fisher Scientific), and then integrated into the binary vector pB7FG2 (Karimi et al., 2002) behind the 35S CaMV promotor via the Gateway^®^ System. The pB7FG2 binary vector also harbors the *bar* gene, which allowed for selection of transgenics using the herbicide Basta. The transformation into maize hybrid Hi-II was performed at the Plant Transformation Facility at Iowa State University (Ames, IA, USA). 35S:*NS1* plants were outcrossed to inbred line B73 three times before use. All maize plants were grown in the Cornell Guterman Greenhouse (conditions: 29.4°C day/23.9°C night; 16 h light/8 h dark; soil type: 1:1 Turface MVP; PROFILE Products).

*Arabidopsis* seeds segregating for the *prs1-1* mutation in the Landsberg *erecta* (L*er*) ecotype were kindly supplied by K. Okada (Matsumoto and Okada, 2001). The *prs1 PRS1-GFP* rescue line was created by transforming *prs1-1* plants with the GFP vector pMDC107 carrying the *PRS1* coding region (AT2G28610) and 3 kb upstream sequences cloned from *Arabidopsis* genomic DNA. The *arf2-12* mutant allele was produced via CRISPR/Cas9 mutagenesis as previously described (Pauwels et al., 2018). WT L*er*, *prs1-1* and *prs1-1 PRS1-GFP* plants were transformed via Agrobacteria (GV3101)-mediated floral dip with the dual sgRNA/Cas9 vector pMR333 obtained from M. Ron (University of California, Davis, CA, USA). Two sgRNAs were designed on CRISPOR (Haeussler et al., 2016) toward the 5′ end of the *ARF2* coding sequence (AT5G62000). The specific sequences were: Protospacer 1 F/R: 5′-ATTGTTTCAATGAAAGGTAATCG/AAACCGATTACCTTTCATTGAAA-3′; Protospacer 2 F/R: 5′-ATTGAATGCACCTGGAACCTCGG/AAACCCGAGGTTCCAGGTGCATT-3′.

BASTA-selected T1 plants were PCR screened using the following primer sequences to identify an 832 bp deletion between the two sgRNA sites, visible via gel electrophoresis. Gene-specific primers used were: 5′-TGGACTACCGAAGCGAGTTT-3′; 5′-TGTGTCGGATGCAGTCAAGG-3′.

The T-DNA insertion in pMR333 also contains a *pOLE-OLE*∼*GFP* (AT4G25140) marker to identify plants carrying the T-DNA insertion by fluorescent seed coat. Seeds from selected T1 plants were thereby screened for the absence of GFP-fluorescence in the seed coat, as a way to select against lines harboring the *CAS9* construct and avoid additional CAS9-mediated mutational activity in the T2 generations and beyond. Lines were progressed until at least the T3 generation and homozygosity was confirmed through PCR and sequencing. All *Arabidopsis* plants were grown in LM111 media (Lambert Peat Moss) under standard long-day conditions (light: 16 h day, 100 µmol; Temperature: 22°C; Humidity: 50%) at the Cornell Agricultural Experiment Station in prototype 45-square foot step-in growth chambers.

### Scanning electron microscopy

*Arabidopsis* cauline leaf axes and flowers were dissected fresh and then flash frozen in slushed liquid nitrogen for scanning electron cryomicroscopy (CryoSEM). Samples were run on a FEI Strata 400S DualBeam Focused Ion Beam scanning electron microscope (FIB/SEM) fitted with a Quorum PP3010T CryoSEM/FIB preparation system. Frozen samples were loaded into a vacuum and briefly sublimated (∼2 min at −80°C followed by 2 min at −70°C) to remove crystalline ice contamination from the transfer process before being sputter coated with gold palladium at 20 mA for 30 s.

### ChIP

ChIP was carried out as previously described (Song et al., 2016) with the following modifications. Approximately 50 meristems, including the P2 and P3 from two-week-old B73 seedlings, were used for each of two biological replicates per antibody; 1 µg anti-NARROW SHEATH 1 rabbit polyclonal antibody (Shimizu et al., 2009, 1/350) was used in the treatment and 1 µg non-specific rabbit IgG was used as a negative control (MAGnify Chromatin Immunoprecipitation System, 49-2024, 1/350). Chromatin was sonicated using the Covaris focused-ultrasonicator. Chromatin was precleared by incubating with anti-rabbit antibody Dynabeads™ before precipitating with Dynabeads incubated with anti-NS1 treatment antibody or nonspecific rabbit IgG negative control. Libraries were prepared using KAPA Hyper Prep Kit (Hoffmann-LaRoche, KK8501) and sequenced on HiSEQ 2500 Rapid Run 2×100 RR Paired End system (Illumina). Reads were aligned with BWA mem settings -M -t aligned to AGPv3. Peaks were called with Macs2 with the following settings: macs2 call peak -t -f BAMPE -g 2060056721 -n -B --call-summits. PAVIS was used to calculate peak distribution across the genome.

### RNA *in situ* hybridizations, immunohistolocalizations and qRT-PCR

Shoot apices from greenhouse-grown two-week-old seedlings were fixed overnight at 4°C in FAA (3.7% formalin, 5% glacial acetic acid and 50% ethanol in water). Tissues were dehydrated at 4°C through a graded ethanol series (50%, 70%, 85%, 95%, 100%) for 1 h each, with three changes in 100% ethanol, and kept in 100% ethanol at 4°C overnight. Tissues were then passed through a graded Histo-Clear (National Diagnostics) series (3:1, 1:1, 1:3 ethanol: Histo-Clear) with three changes in 100% Histo-Clear; all changes were 1 h each at room temperature. Samples were then embedded in Paraplast^®^Plus (McCormick Scientific), sectioned and hybridized using antisense digoxygenin-labeled RNA probes as previously described (Johnston et al., 2014a).

Hybridization probes for *NS1* (GRMZM2G069028) were prepared as previously described (Nardmann et al., 2004). Gene-specific primers were used to prepare 761 bp-long *in situ* hybridization probes for *ARF25* (GRMZM2G116557): 5′-GATGACAGTCGTCACCGTCT-3′; 5′-TTAGGAACCAAACCACCAGG-3′.

Immunolocalizations were carried out as previously described (Boutte et al., 2006; Lee et al., 2009) using an *Arabidopsis* PIN1 (gift from J. Traas, ENS de Lyon, France) antiserum diluted 1:300 or a 1:350 dilution of affinity-purified rabbit anti-NS1 antiserum (Shimizu et al., 2009), and the Alexa Fluor 488-conjugated secondary antibody (Life-Technologies, 1/500).

For qRT-PCR analyses of gene-specific auxin and cytokinin responses, root excised 14-day-old B73 maize seedlings were incubated with IAA or kinetin, which was first dissolved in 1 M KOH then diluted to a working concentration of 0.1 μM at pH 5.8. Control samples were cultured in soil treated with water containing an equimolar concentration of KOH. Gene-specific primers (below) were designed for use with SYBR-Green (Quanta) in qRT-PCR as previously described (Zhang et al., 2007). Three biological replicates were examined; data are presented using the 2^−ΔΔct^ method (Livak and Schmittgen, 2001) with threshold values normalized to accumulation of each transcript after control treatment as described using Bio-Rad iQ5 Version 1.0 software (Zhang et al., 2007). The gene-specific primers used were: *ZmNS1*-GRMZM2G069028–5′-ATGGAGGTGGAGCTGGGTTA-3′, 5′-CACAGATCAGTGCTCCATTGCATCTGTG-3′; *ZmHK1*-GRMZM2G069028–5′-GGCTCGACAACTGCCGAGTAC-3′, 5′-GTCGTTCCCACTACCAATCTGGAG-3′; *ZmARF5*-GRMZM2G035405–5′-GCTATCACGAGCTCCGTAGG-3′, 5′-CGGTCGACGAATACAAGCTG-3′; *ZmRR7*-GRMZM2G096171–5′-CTCGCACTACTTCCAGTTCCTCCTC-3′, 5′-GACGGAGCCATTGGACCATCTG-3′.

### Microscopic imaging

Light microscopic imaging of sectioned samples was performed as previously described (Johnston et al., 2014a). Confocal imaging was performed as previously described (Shimizu et al., 2009). CT imaging was performed as previously described (Johnston et al., 2014b).

### Laser microdissection, library preparation and sequencing

Two-week-old seedlings of *ns1* mutants and wild-type seedlings from the *ns* 1:1 line (described above) were dissected and fixed and embedded for LM-RNA-seq) as previously described (Scanlon et al., 2009). The marginal tips of P2 and P3 leaves were targeted for microdissection from 10 µm transverse serial sections (Fig. 2D,E) based on the localization of NARROWSHEATH1 protein in immunohistological sections (Fig. 1E; Fig. S1) using the Positioning and Ablation with Laser Microbeams system (PALM; Microlaser Technologies). RNA extraction was performed according to the manufacturer's instructions using Arcturus™ PicoPure™ RNA Isolation Kit and RNA amplification using the Arcturus™ RiboAmp™ HS PLUS Kit (Thermo Fisher Scientific). Libraries were prepared using the NEBNext UltraTM RNA Library Prep Kit for Illumina, and sequenced using NextSeq 500 75 Single End.

### RNA-seq alignment, counting and normalization

Illumina adapter sequences were trimmed using Trimmomatic v0.39. Reads were aligned to B73 genome RefGen V3 with HiSAT2 (Kim et al., 2015) and counted with HTSeq (Anders et al., 2015). Raw counts were normalized using R package edgeR v3.20.9. Differential expression was calculated using R package DESeq2. The raw ChIP-seq data and RNA-seq data are available at the NCBI Bioproject number PRJNA633509.

## Supplementary Material

Supplementary information
