# Supplementary material for: Plant homeodomain proteins provide a mechanism for how leaves grow wide
Source: Development. 2020 Oct 21;147(20):dev193623. doi: 10.1242/dev.193623 (PMC7595687; doi:10.1242/dev.193623)
Supplement: Supplementary information [file develop-147-193623-s1.pdf]

Table S1. Genomic targets bound by NARROW SHEATH1.

Click here to Download Table S1

Table S2. RNAseq analyses of laser microdissected margins from P2 and P3 leaf primordia in wild type and narrow sheath mutants.

Click here to Download Table S2

Table S3. Genes bound and modulated by NARROW SHEATH1.

Click here to Download Table S3

Table S4. Gene targets bound and repressed by NARROW SHEATH1.

Click here to Download Table S4

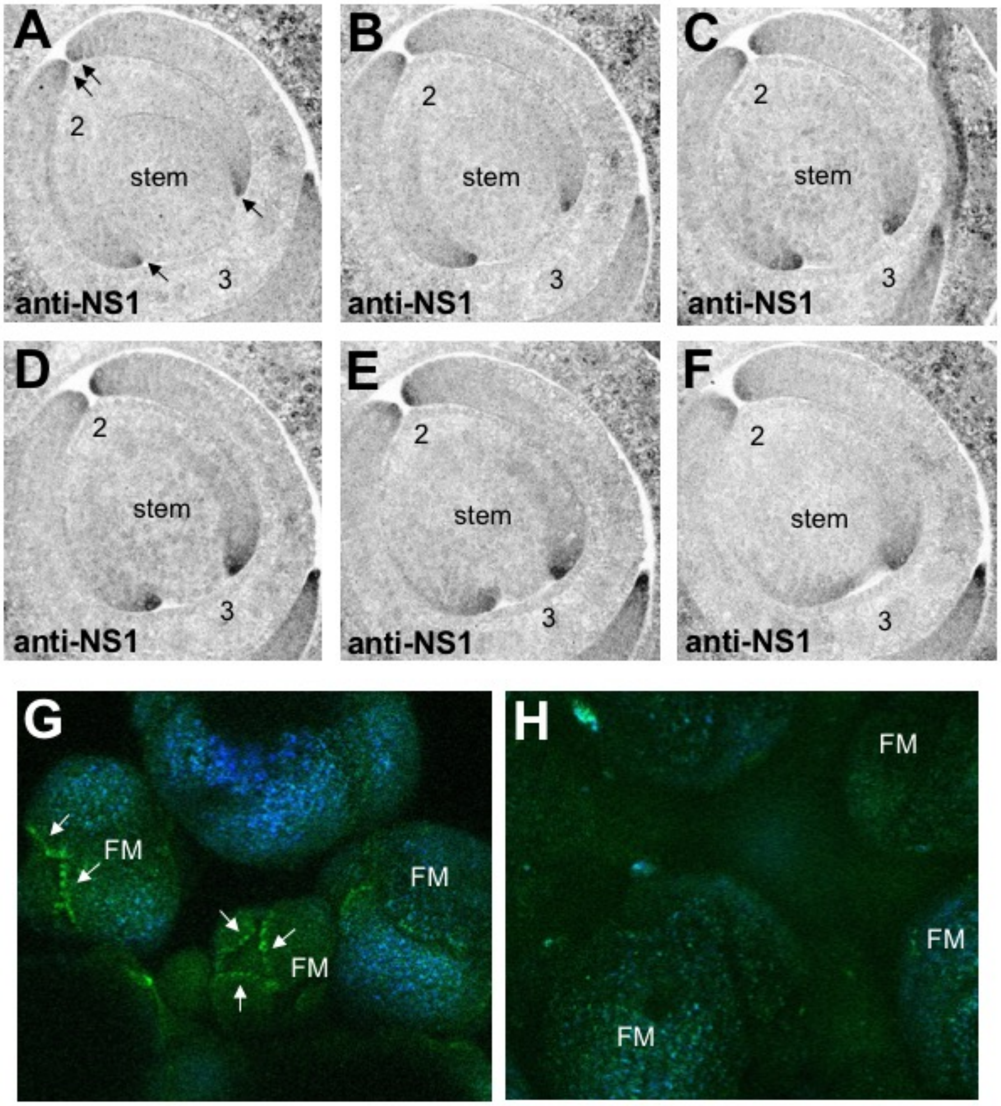

Fig. S1. NS1 in maize and PRS1~GFP in Arabidopsis localize to the margins of developing leaves and sepals, respectively. (**A-F**) Immunohistolocalization of NS1 protein accumulation in serial, basipetal, transverse 10um sections of two-week old seedlings, using the anti-NS1 antibody. NS1 protein localizes to the margins of P2 and P3 leaves (arrows). (**G**) PRS1~GFP fusions show PRS1 protein localization at the sepal margins in early stage flowers. (**H**) Non-transgenics plants show no GFP accumulation in the margins of early stage flowers.

Development • Supplementary information

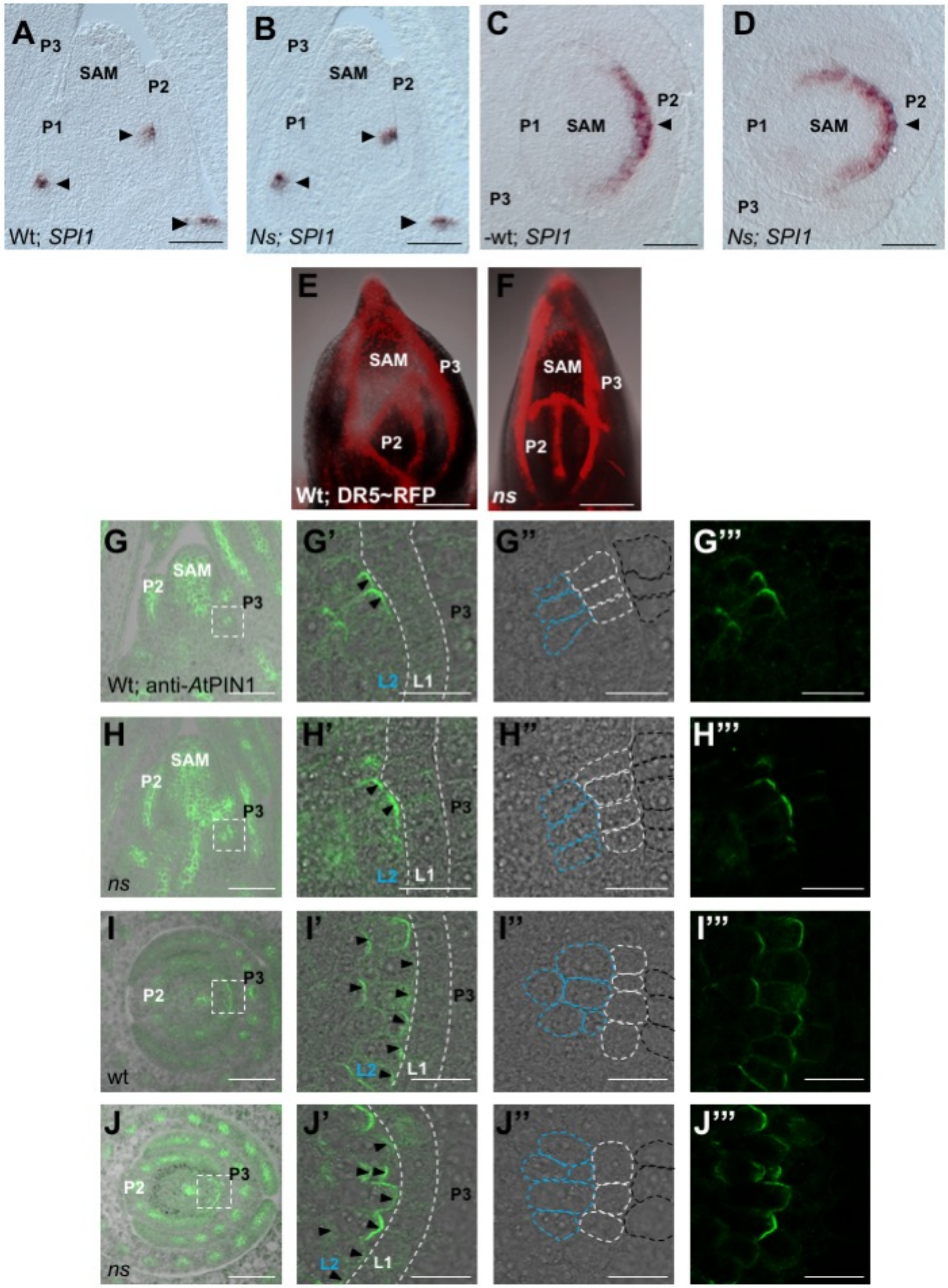

Fig. S2. *NS1* functions downstream of auxin biosynthesis, auxin response, and auxin transport. (**A-D**) *SPARSE IINFLORESCENCE1*(*SPI1*) accumulation in wild type (Wild type; **A, C**) and *narrow sheath* (*ns;* **B, D**) longitudinal (**A, B**) and

transverse (**C, D**) sections. P1, P2, P3 designate successive leaf primordia where P1 is the youngest leaf closest to the shoot apical meristem (SAM); arrowheads indicate corresponding margin domains of leaf primordia. *SPI1* transcript accumulates in a ½ ring around the SAM, corresponding to the margin regions of the P1 primordium, and persists in the margin domains of older leaf primordia in both wild type and *ns*. (**E-F**) DR5~RFP localization in wild type (**E**) and *ns* mutant (**F**) shoots containing the SAM and three leaf primordia. DR5~RFP signal marks the margins and midrib region of P2 and P3 primordia in both WT and *ns* mutant primordia. (**G-J**) PIN1 dynamics during leaf margin initiation. PIN1 immunolocalization (green signal) in wild type (**G, I**) and *ns* (**H-J**) longitudinal (**G-H**) and transverse (**I-J**) sections of seedling shoot apices. Boxed regions in (**G-J**) show localization of PIN1 (green signal) in the L2 layer of the emerging marginal regions of P2 staged leaf primordia; insets show enlargements of boxed areas. P1, P2, P3 designate successive leaf primordia as above. Arrows and black arrowheads indicate direction of auxin transport as inferred from PIN1 localization. In the emerging marginal domain of P2 leaf primordia, the direction of auxin transport is from the inner cell layers (L2), to the outer cell layer (L1) in both wild type and *ns.* Dashed lines in **H'-J'** indicate L1 layer. Individual cells are outlined in brightfield images **H''-G''**; white = L1 layer cells, blue = L2 layer cells, black = P3 cells. Images in **G-G'', H-H'', I-I'', and J-J''** have brightfield overlay, images **G'''- J'''** are dark field. Scale bars: 100μm in A-F; 20μm in G-J.

Development • Supplementary information

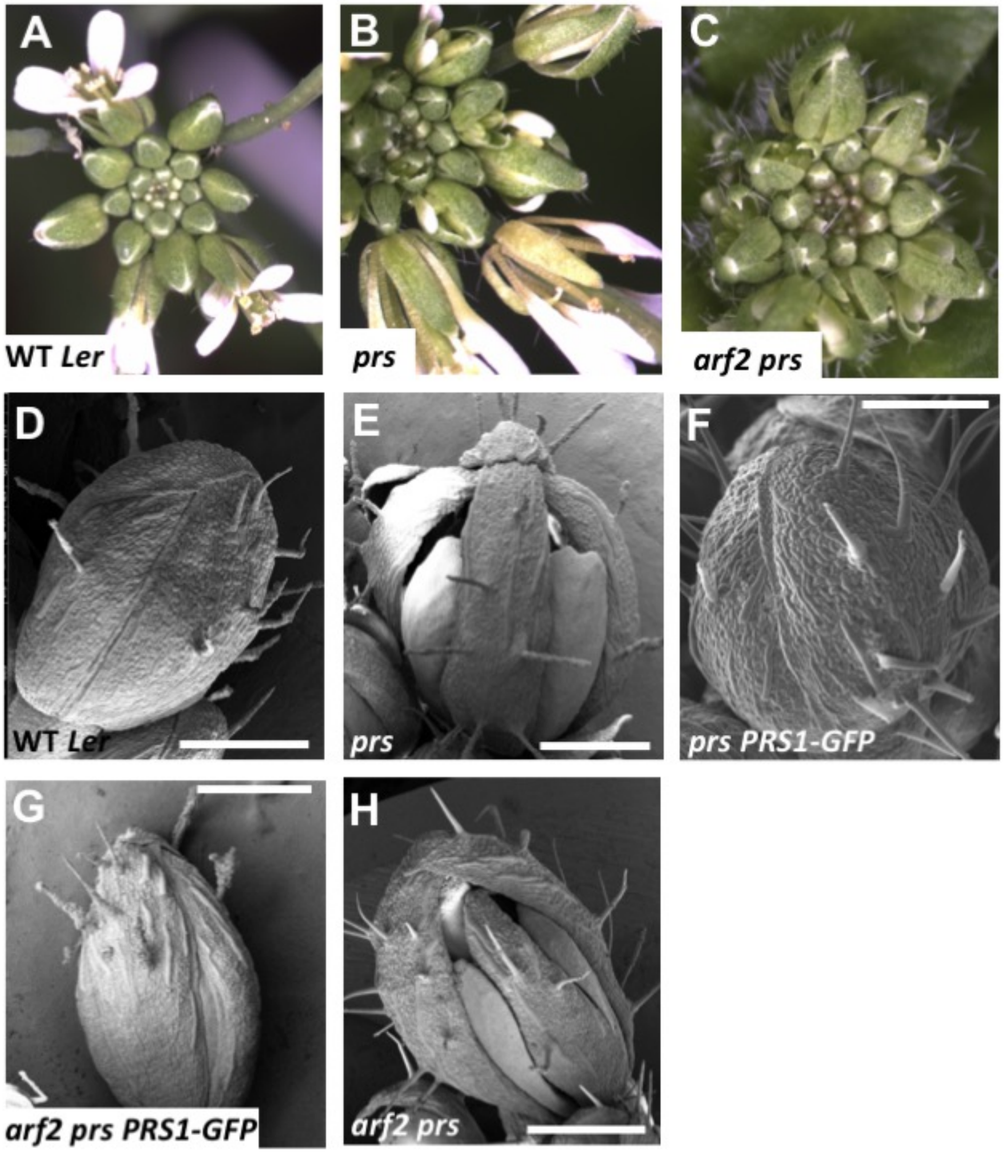

Fig. S3. *arf2* mutations fails to rescue the *prs* mutant sepal phenotype. Whole Arabidopsis inflorescence for (**A**) Wild type Landsberg *erecta* (WT *Ler*), (**B**) *prs* and (**C**) *arf2-12 prs* genotypes. Cryo-scanning electron micrograph (CryoSEM) of single Arabidopsis flowers for the genotype: (**D**) WT *Ler*, (**E**) *prs*, (**F**) *prs PRS1-GFP*, (**G**) *arf2-12 prs PRS1-GFP*, and (**H**) *arf2-12 prs*. Scale bars: 500 μm.

Development • Supplementary information
